# Improving our forecasts for trachoma elimination: What else do we need to know?

**DOI:** 10.1371/journal.pntd.0005378

**Published:** 2017-02-09

**Authors:** Amy Pinsent, Manoj Gambhir

**Affiliations:** Department of Epidemiology and Preventive Medicine, Faculty of Medicine, Nursing and Health Sciences, Monash University, Melbourne, Victoria, Australia; RTI International, TANZANIA, UNITED REPUBLIC OF

## Abstract

The World Health Organization (WHO) has targeted trachoma for elimination as a public health concern by 2020. Mathematical modelling is used for a range of infectious diseases to assess the impact of different intervention strategies on the prevalence of infection or disease. Here we evaluate the performance of four different mechanistic mathematical models that could all realistically represent trachoma transmission. We fit the four different mechanistic models of trachoma transmission to cross-sectional age-specific Polymerase Chain Reaction (PCR) and Trachomatous inflammation, follicular (TF) prevalence data. We estimate 4 or 3 parameters within each model, including the duration of an individual’s infection and disease episode using Markov Chain Monte Carlo. We assess the performance of each models fit to the data by calculating the deviance information criterion. We then model the implementation of different interventions for each model structure to assess the feasibility of elimination of trachoma with different model structures. A model structure which allowed some re-infection in the disease state (Model 2) was statistically the most well performing model. All models struggled to fit to the very high prevalence of active disease in the youngest age group. Our simulations suggested that for Model 3, with annual antibiotic treatment and transmission reduction, the chance of reducing active disease prevalence to < 5% within 5 years was very low, while Model 2 and 4 could ensure that active disease prevalence was reduced within 5 years. Model 2 here fitted to the data best of the models evaluated. The appropriate level of susceptibility to re-infection was, however, challenging to identify given the amount and kind of data available. We demonstrate that the model structure assumed can lead to different end points following the implementation of the same interventions. Our findings are likely to extend beyond trachoma and should be considered when modelling other neglected tropical diseases.

## Author summary

Trachoma is the world’s leading infectious cause of blindness. Mathematical models are used by researchers to examine the spread of infectious diseases and understand how they can be controlled. Such models are developed based on the natural history of infection. For trachoma we identify four different model structures which could all represent the natural history of trachoma infection. We fit each of the models to infection and disease prevalence data for 3 different age groups. We find that one of the models is able to fit the data better than others, however some factors about the model are difficult to identify due to limited data. The ease of eliminating disease within a community assuming the same interventions varied depending on the model structure assumed. Our results highlight that some models of trachoma fit to infection and disease data better than others, but that more data is needed to identify more specific aspects of the model structure. In addition we show that different model structures may give different results in terms of the effort required to control trachoma transmission.

## Introduction

Trachoma remains the world’s leading infectious cause of blindness. 200 million people are reported to be at risk of infection, across 42 endemic countries [1]. The causative agent of infection is the bacterial pathogen *Chlamydia trachomatis* [2]. The World Health Organization through the Alliance for the Global Elimination of Trachoma by 2020 (GET2020) is aiming to eliminate trachoma as a public health problem by 2020. Two goals have been developed to assist endemic countries striving to achieve the elimination of trachoma as a public health problem. The first goal aims to reduce the prevalence of Trachomatous inflammation, follicular (TF) in children aged 1–9 years, to less than 5% by 2020. Mathematical modelling has been successful in helping to formulate guidelines for the ongoing surveillance and control of a range of infectious diseases including malaria [3], onchocerciasis [4], lymphatic filariasis [5] and soil-transmitted helminths [6]. Furthermore, mathematical models can be used to provide guidance on suggested timelines to elimination or control, for a given set of initial conditions and available interventions. However, to generate informative and accurate predictions, models need to be informed by high quality epidemiological data, particularly in terms of the duration of infection and disease, as it is these states which are detectable through diagnostic tests.

For trachoma, the control guidelines are based on the disease which occurs as a consequence of infection with *C. trachomatis* bacteria. Therefore, guidelines are based not on monitoring the causative agent of infection directly, but the longer-term disease associated with it [7]. It is understood that individuals can remain TF positive with detectable disease for far longer than they are Polymerase Chain Reaction (PCR) positive. Despite this, estimates of the duration of PCR detectable infection and the duration of disease are not commonly available and are rarely estimated [8]. Nonetheless, a good understanding of the time spent in these states is vital if accurate model projections on time to elimination of trachoma are to be developed. For example, in the absence of on-going sustained transmission in an endemic region, if one assumed that the duration of a disease episode was less than 1 month (as estimated for individuals 15 years or older in the community [8]) the expected time to reach the < 5% elimination threshold would be much shorter than if the assumed duration of disease was 2 years; thus, the assumed rate of recovery from disease is likely to have a large impact on the expected time to reach elimination targets.

Mathematical models are developed and informed by the natural history of infection. For trachoma, the Susceptible, Infected, Susceptible (*SIS*) model structure has most commonly been used [9–15] where individuals in the *S* state are susceptible to infection and those in the *I* state are infected and infectious and, thus, are PCR detectable. Such models are fitted to PCR data collected during clinical trials of trachoma treatment [9, 10, 12]. However, current control guidelines are based on disease, not PCR detectable infection. Therefore, if models are to be informative in terms of whether the guidelines on TF prevalence will be achieved, it may be desirable to capture the dynamics of both PCR and TF positivity, although this has rarely been done [16, 17]. The lack of modelling work in this field is likely to have been exacerbated by the limited longitudinal population level data that measure both PCR and TF positivity across multiple age groups, particularly following the implementation of interventions.

In addition to the *SIS* compartmental model structure, several other variant structures may also be considered appropriate given the natural history of trachoma infection [18–21]. It has been reported for other infectious diseases that the structure of the model assumed can impact the estimated effort required to control and eliminate that disease [22–24]. Therefore, when modelling the transmission of trachoma to make projections on the feasibility of elimination, it is important to select not only the most parsimonious and statistically appropriate model, but also to understand how the assumed model structure may impact elimination projections.

Here we compare four different model structures which could realistically all represent the natural history of trachoma infection as understood by epidemiologists through the interpretation of experimental data. We statistically fit each model to cross-sectional data on bacterial load, PCR and TF prevalence for three different age groups. We then evaluate the performance of each model structure using the Deviance Information Criterion (DIC). With the parameter estimates obtained from each of the best performing models we assess the feasibility of and time to elimination, to understand if and how they differ.

## Materials and methods

### Data

We used cross-sectional data on PCR and TF prevalence in individuals aged 1–4, 5–14 and 15 years or older, collected from a hyperendemic community in Tanzania at one point in time, prior to the roll out of trachoma interventions [25]. Data on the mean bacterial load by age group were available from [25]. Data on age-specific bacterial load, PCR and TF prevalence were used to fit each of the models evaluated.

### Model structure

**Fig 1:**
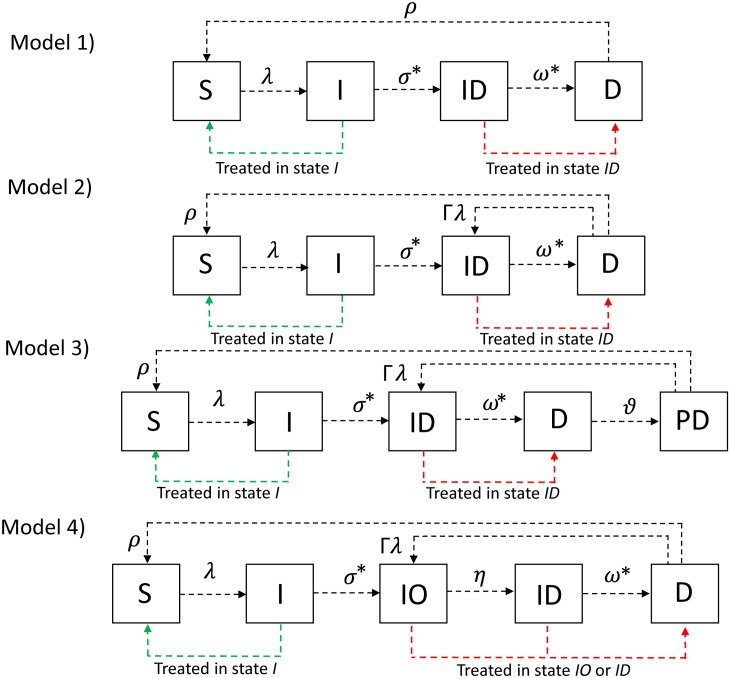
Schematic of the different model structures evaluated. A) Represents Model 1, here individuals in the *D* are 100% immune to re-infection. B) Represents Model 2, where individuals in the *D* state can be re-infected. C) Represents Model 3 [18], where individuals are 100% immune to re-infection in the *D* state but can be re-infected once they progress to the *PD* state. D) Represents Model 4 where individuals in the *IO* state spend a period of time only PCR positive, then progress to *ID* where they are PCR and TF positive. Coloured arrows illustrate how treatment within each model structure is implemented. Individuals who are infected but not infectious when treated return to the *S*_*i*_ class they were in before they were infected (indicated by the red arrow), hence no immunity is acquired as a result of infection. For those in the *ID*_*i*_ or *IO*_*i*_ class who are successfully treated they progress to the *D* (indicated by the green arrow) and were assumed to acquire immunity as a consequence of the infection they experienced. Treatment was assumed to not impact those in the disease only states. The (*) around parameters indicate that the minimum rate of recovery of these parameters was estimated.

We evaluate 4 different plausible natural histories of infection [9, 11, 13, 26] and disease [7, 16, 18, 19] that may occur following exposure to trachoma. The model structures we evaluate highlight the clinical and epidemiological observations made in the field and laboratory [19, 21, 27, 28]. The first model, Model 1, follows the structure represented in Fig 1a. Here susceptible individuals (*S*) become infected at a rate *λ*, they incubate infection in the (*I*) state, and progress at a rate *σ* to the infected infectious state(*ID*) where they test PCR positive and TF positive. Individuals leave *ID* at a rate *ω* and progress to the disease only state (*D*), where they are only TF positive, and recover from the disease only state at a rate *ρ* and return to the susceptible state (*S*).

For Model 2 we assume the same structure as Model 1 (Fig 1b), however we do not assume individuals in the *D* state are 100% immune to re-infection [19]. Instead we explore 3 levels of susceptibility to re-infection (Γ): 20%, 50% and 80%. All other transitions are the same as described in Model 1.

In Model 3 (Fig 1c) we evaluate the structure previously postulated by Shattock *et al* [18]. Here, the first 3 states are identical to those described in Model 1 and 2. However, for Model 3 we split the duration of time spent in the *D* state across two compartments. In the *D* state, individuals are immune to re-infection. They then progress to the *PD* state at a rate *γ* [19]. In this state individuals are susceptible to re-infection with the same susceptibility levels described in Model 2.

Model 4 (Fig 1d), we introduce an additional infected state, the *IO* state, which comes after the incubating state, where individuals are not infectious (*I*), but prior to the *ID* state. In the *IO* state individuals have a PCR detectable infection, but are not yet TF positive. From here individuals progress to *IA* at a rate *η* where they are PCR and TF positive, individuals recover from their infection and progress to the *D* state, where they are only TF positive, but as with Models 2 and 3 individuals could experience re-infection in the (*D*) state with the same susceptibility levels described in Models 2 and 3 [19].

All models follow the ‘ladder of infection’ structure [11, 16, 26], whereby each subsequent infection leads to improved immunity following re-infection. In all 4 model structures we reflect improving immunity as an increase in the rate of recovery from infection and disease episodes, in addition to a reduction in infectivity with each successive infection. We assume that the infectivity of an individual is proportional to their bacterial load. In the model we reflect declines in bacterial load with repeated infection as reductions in an individual’s infectivity to others. This represents a trend in agreement with the data from trachoma endemic communities in which the bacterial load decreases with age [25, 29, 30]. For each model structure (A-D, Fig 1) we have two sub-variants, the 4-parameter and the 3-parameter versions. These models pertain to two alternative sets of assumptions about how bacterial load and infectivity decline with consecutive infections. We assume for the 4 parameter model that infectivity is proportional to bacterial load and therefore declines exponentially with the number of prior infections. For the 3 parameter model we assume that infectivity declines linearly with the log of the bacterial load i.e. linearly with the number of prior infections. We chose exponential functions as fairly flexible low-parameter functions that, for the rates of recovery, would accomplish the goal of a) rising from an initial value–for no and low numbers of infections–to b) saturating at a high value for high numbers of infections. We note that we also tested the use of a log-logistic function instead of an exponential, however it was no better performing than the exponential function.

Additional detail on the model parameters and state variables are presented in Table 1. Details on the immunity functions and mathematical equations for each model are presented in S1 File.

**Table 1:** State variables, parameters definitions and values used in the model. Where two numbers are listed for *ψ*, they indicate the values used for TF 40% and 20% communities.

Name	Definition	Value	Units	Source
*S*_*i*_	Susceptible individuals		Number	
*I*_*i*_	Infected but not infectious		Number	
*ID*_*i*_	Infected and Infectious (PCR and TF +ve)		Number	
*D*_*i*_	Diseased and not infectious (TF +ve)		Number	
*PD*_*i*_	Partially diseased can be re-infected (TF +ve)		Number	
*IO*_*i*_	Infected and infectious (PCR +ve)		Number	
*β*	Transmission rate parameter	Estimated	Proportion	
*ϵ*	Degree of random mixing in the population	0.5	Proportion	[11]
*c*	Coverage level of treatment	80%	Percentage	
*e*	Efficacy of treatment	85%	Percentage	[12, 31]
*N*_*infs*_	Maximum number of infections before immunity saturates	100	Number	[11]
*N*	Total number of individuals in the population	6000	Number	
*α*	Infectivity of an individual proportional to the log of their bacterial load	0–1	Proportion	[11]
*ρ*_1_	Minimum rate of recovery from active disease after 1st infection	Estimated	Day^−1^	[8, 11]
*ρ*_100_	Maximum rate of recovery from active disease after 100th infection	1/7	Day^−1^	[8, 11]
*ω*_1_	Minimum rate of recovery from 1st infection	Estimated	Day^−1^	[8, 11]
*ω*_100_	Maximum rate of recovery from 100th infection	1/77	Day^−1^	[8, 11]
*θ*	Rate of change of the recovery from disease rate per infection	0.30	Proportion	[8, 11]
*ϕ*	Rate of change of the recovery from infection rate per infection	0.45	Proportion	[8]
*π*	Rate of change of infectivity rate per infection	Estimated	Proportion	[8, 11]
*ψ*	Non-linear power term	1.2, 1.4	Number	[9]
*ζ*_1_	Rate of recovery from *PD* with 1st infection	1/134	Day^−1^	[8, 18]
*ζ*_100_	Rate of recovery from *PD* with 100th infection	1/7	Day^−1^	[8, 18]
*η*_1_	Rate of recovery from *IO* with 1st infection	1/77	Day^−1^	[8]
*η*_100_	Rate of recovery from *IO* with 100th infection	1/38	Day^−1^	[8]
*σ*	Rate at which infected individuals become infectious	1/14	Day^−1^	[8]
*λ*_*a*_	Age-specific force of infection	Calculated		
*v*_2_	Non-linear constant term	2.6	Number	[9]
Γ	Susceptibility to re-infection in the disease state	0, 0.20, 0.50, 0.80	Proportion	[18]

### Parameter values

All parameter values and definitions are provided in Table 1. We assume that the mean minimum duration of an infection episode was 10 weeks and the duration of a disease only episode was 1 week (Table 1), the same as those estimated for the oldest age group in Grassly et al [8]. We take estimates from the oldest age group to parameterise the minimum duration of an infection and disease episode. Immunity to trachoma is thought to develop through repeated infections. Therefore as those in the highest age group are most likely to have experienced the highest number of infections, we assumed that they would have the highest levels of immunity. It is inherently challenging to estimate immunity functions [32] and, given only 3 data points were available, the true values of any immunity parameters were likely to be unidentifiable. As such, exponential increases in the rate of recovery from infection and disease, with the number of prior infections experienced by an individual, were informed by Grassly et al [8]. Age-specific estimates of the duration of infection and disease, and were fixed for the purposes of model fitting.

### Model fitting

Each of the four model structures evaluated were fitted as 3 and 4 parameter models to the data. An additional factor: the relative susceptibility of the diseased, non-infected state for new infections was varied. One value was used for Model 1, and 3 different values for each model structure 2, 3 and 4. Therefore a total of 10 different models were fitted for each parameterisation of the model structures. For the 4 parameter models we estimated the transmission rate parameter *β* per *day*^−1^ for the data, the duration of an individual’s first infection and disease episode in the *ID* and *D* states, and the rate at which infectivity changed with each successive infection for the bacterial load function (Table 1). For the 3 parameter model we estimated the first three parameters listed in the 4 parameter model, but assumed a constant linear decline in the log load of an individual’s bacterial load with each successive infection, thus, in the 3 parameter model we did not estimate the rate of increase in improved immunity with re-infection.

Parameter estimation was performed using Markov Chain Monte Carlo (MCMC). The chains were run for 10,000 iterations. The Robbins-Munro algorithm was implemented as part of the adaptive stage of the MCMC-Metropolis Hastings algorithm, to ensure the proposal distributions were adaptively tuned ensuring efficient exploration of the posterior [33]. Selection of the most parsimonious model and fit of each model to the data was assessed using the DIC [34], therefore we assumed our posterior distribution was approximately multivariate normally distributed. Fits to the data for each model are presented in Table S2 in S1 File. Estimates from the 4 parameter model are provided in Table S3 in S1 File and estimates from the 3 parameter model are provided in Table S4 in S1 File. Uninformative uniform priors were specified for all parameters. MCMC diagnostics are presented in Table S5 in S1 File. We calculate the Gelman-Rubin statistic for 2 MCMC chains to assess convergence [35] and the Effective Sample Size (ESS) for each model fit.

### Modelling different interventions

For each model structure (described above) we performed simulations to assess the potential impact of Facial cleanliness and Environmental improvements (F and E) within the community, along with the implementation of mass drug administration (MDA). All simulations were started from endemic equilibrium. For communities with greater than 20% TF, 5 annual rounds of MDA were performed, and for those with TF 20% or less we performed 3 rounds of annual MDA. We also assessed the possible impact of F and E to reduce transmission. The true impact of F and E remains poorly quantified [36], therefore we consider a range of reductions in transmission that may be possible (between 0–50%). *β* was assumed to decline exponentially over the intervention period, to model an increasing uptake of transmission reduction interventions in the community over time. We model changes in *β* as an instantaneous drop when each annual round of MDA is performed as we assumed intensified health promotion activity would be conducted when MDA was distributed. Reductions in transmission which were only considered to occur through the implementation of F&E and were assumed to be maintained following the cessation of treatment.

For each model structure we assessed the time taken and the feasibility of reducing TF prevalence to less than 5% within the community in children under 10 years old. A constant level of treatment coverage (80%) between each round and across model comparisons was assumed, along with a fixed treatment efficacy of 85% [12, 31]. A schematic of the movement of individuals between compartments following treatment is illustrated in Fig 1. We assess the sensitivity of findings on the feasibility of elimination for the different model structures to variation in 6 different fixed parameters, these are: treatment efficacy, duration of first infection and disease episodes, maximum rate of recovery from infection, maximum rate of recovery from disease and the degree of age mixing in the population. These were assessed across both transmission settings and all levels of transmission reduction due to F and E.

## Results

**Fig 2:**
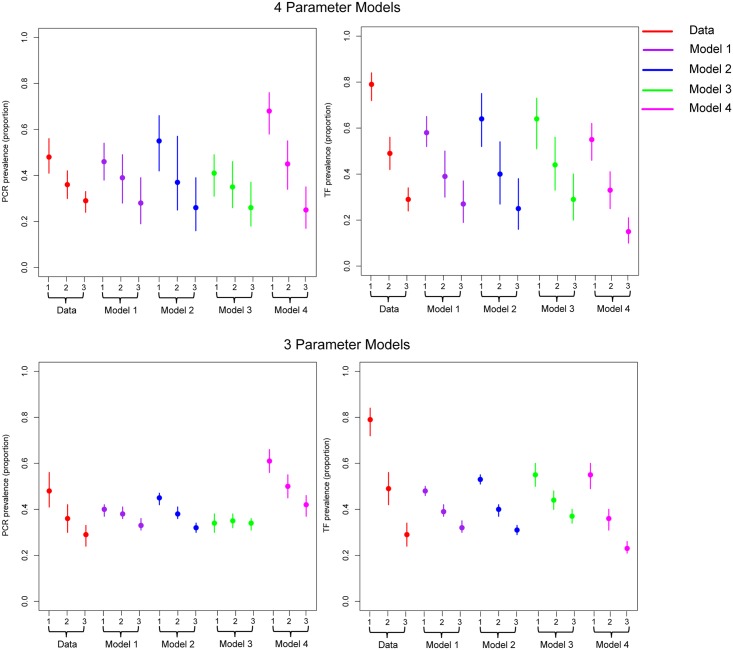
Estimates from the best performing models of the age-specific PCR and TF prevalence. Estimates of age-specific TF and PCR prevalence from statistically the best performing models for each structure evaluated. Data is shown in red, Model 1 results are shown in purple, Model 2 results are shown in blue, Model 3 results are shown in green, and Model 4 results are shown in pink. The first row shows PCR and TF fits from the 4 parameter models. The second row shows PCR and TF fits from the 3 parameter model. Lines around each model’s point estimate are the 95% credible intervals.

None of the model structures evaluated captured the very high reported prevalence of TF in children aged 1–4 years (Fig 2) but they were able to capture TF prevalence in the older two age groups well (Fig 2). Equally, all models fit the age-specific PCR data well. Across all structures explored it was not possible to capture the high average bacterial loads reported in young children, assuming the exponential function for the development of immunity with bacteria load, but this was possible when assuming a linear decline (Table S4 and Figure S1 in S1 File). Although, assuming the linear model typically resulted in higher estimates in the bacterial load in older age groups, not seen in the data (Table S4 and Figure S1 in S1 File).

In general across the 3 and 4 parameter models, predicted age-specific prevalence of infection and disease were lower for Model 3 in comparison to Model 4 (Fig 2). Typically with Model 4 the estimated PCR prevalence was too high for all age groups in comparison to the data. The higher level of PCR prevalence obtained by Model 4 reflects the overall longer duration of PCR detectable infection for Model 4 in comparison to Model 3. Thus, we would expect that these models’ equilibrium age-specific prevalence levels would differ from one another. Model 2 and 3 had similar predicted values of age-specific TF prevalence but predicted PCR prevalence was lower for Model 3 in comparison to Model 2 (Fig 2). This may be because the duration of the disease only episode in Model 3 was longer than Model 2, therefore it takes longer for individuals to get re-infected and test PCR positive under Model 3 in comparison to Model 2. The estimated age-specific prevalence for infection and disease was lower for Model 1 in comparison to Model 2, resulting in the fits from Model 2 more closely aligning with the data than the fits from Model 1. This difference may reflect the importance of allowing some susceptibility to re-infection on individuals in the TF only state.

The mean estimated duration of infection and disease periods for an individual’s first infection were roughly comparable across all models when 3 or 4 parameters were estimated (Table S2 and Table S3 in S1 File). However, the estimated value of *β* (day^−1^) across the different structures evaluated varied substantially, this was true for both the 3 and 4 parameter models. Estimates from Model 3 provided the largest estimated values of *β*. This is likely to be because this model structure includes two states that do not contribute to the overall force of infection, thus in order to fit to the high level of infection and disease a very high value of *β* was needed relative to the other models evaluated. Within a given model structure estimates of *β* were less affected by the assumed level of susceptibility to re-infection in the TF only state for Model 4. This may be due to the longer duration of infection reflected under this structure in comparison to the others. This longer duration of infection also meant that the estimated value of *β* for Model 4 was the lowest of all structures evaluated.

The 4 parameter version of Model 2 with varying levels of susceptibility to re-infection provided the most parsimonious fit to the data, according to the DIC (Fig 2, Table S2 in S1 File). For Model 4, assuming high levels of susceptibility to re-infection in the disease state was comparable in performance to Model 3. DIC scores for Models 1, 2, 3 and 4 were: 1942.44, 1928.04, 1949.79 and 1950.60 for each model respectively. For the three parameter model (Table S3 in S1 File) assuming a linear decline in bacterial load, Model 2 also had the lowest DIC score out of the four structures assessed. These findings suggest that while there is statistical evidence to suggest that re-infection in the disease only state is important, with the current data available, it is not possible to identify which level of susceptibility is most appropriate.

### Simulating interventions under different model structures

**Fig 3:**
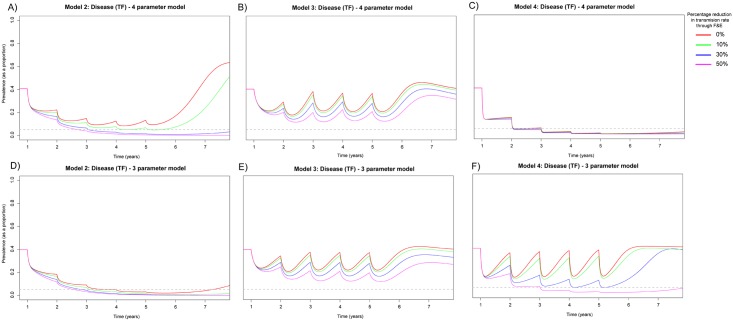
Prevalence of TF in 0–9 year olds when MDA has been applied for 5 annual rounds for 5 years within a community with 40% TF prevalence. A) Model 2 assuming 20% susceptibility to re-infection in the *D* and assuming an individual’s infectivity decayed exponentially with each successive infection. B) Model 3 assuming 20% susceptibility to re-infection in the *PD* and assuming an individual’s infectivity decayed exponentially with each successive infection. C) Model 4 assuming 80% susceptibility to re-infection in the *D* and assuming an individual’s infectivity decayed exponentially with each successive infection. D) Model 2 assuming 20% susceptibility to re-infection in the *D* and assuming a linear decline in infectivity with each successive infection. E) Model 3 assuming 20% susceptibility to re-infection in the *PD* and assuming a linear decline in infectivity with each successive infection. F) Model 4 assuming 80% susceptibility to re-infection in the *D* and assuming a linear decline in infectivity with each successive infection. For the 4 and 3 parameter versions of Models 2–4 (A–F) we considered variable reductions in the transmission rate *β* that may be achievable through facial cleanliness and environmental improvements (F&E). We consider instantaneous non-linear declines in *β* across the 5 year intervention period. We considered maximum reductions in *β* over the 5 year intervention period to be 0, 10, 30 or 50% from the initial value used.

**Fig 4:**
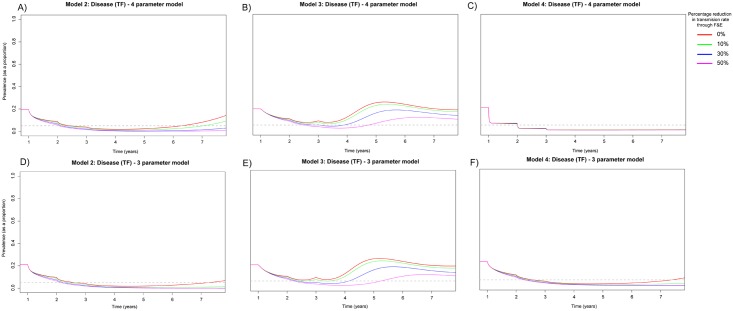
Prevalence of TF in 0–9 year olds when MDA has been applied for 3 annual rounds for 3 years within a community with 20% TF prevalence. A) Model 2 assuming 20% susceptibility to re-infection in the *D* and assuming an individual’s infectivity decayed exponentially with each successive infection. B) Model 3 assuming 20% susceptibility to re-infection in the *PD* and assuming an individual’s infectivity decayed exponentially with each successive infection. C) Model 4 assuming 80% susceptibility to re-infection in the *D* and assuming an individual’s infectivity decayed exponentially with each successive infection. D) Model 2 assuming 20% susceptibility to re-infection in the *D* and assuming a linear decline in infectivity with each successive infection. E) Model 3 assuming 20% susceptibility to re-infection in the *PD* and assuming a linear decline in infectivity with each successive infection. F) Model 4 assuming 80% susceptibility to re-infection in the *D* and assuming a linear decline in infectivity with each successive infection. For the 4 and 3 parameter versions of Models 2–4 (A–F) we considered variable reductions in the transmission rate *β* that may be achievable through facial cleanliness and environmental improvements (F&E). We consider instantaneous non-linear declines in *β* across the 5 year intervention period. We considered maximum reductions in *β* over the 3 year intervention period to be 0, 10, 30 or 50% from the initial value used.

Across both transmission settings infection was more likely to re-emerge if the infectivity was assumed to decline exponentially (Figs 3, 4a–4c). However, in general, this functional form led to an overall better fit to the cross-sectional data (Table 1). This somewhat counterintuitive effect with exponentially declining infectivity (explored in [37]) results in the reproduction number associated with the full model increasing with each subsequent treatment round of MDA. This is as a result of the concentration of infectivity increasing with multiple rounds of MDA, as a higher number of individuals in the population have experienced fewer infections, resulting in individuals aggregating at higher infectivities as their progress along the ‘ladder of infection’ is slowed or halted due to MDA. When assuming an exponential decline in infectivity it was not possible to eliminate disease from the community with 40% TF prevalence with Models 2 or 3, even with annual MDA for 5 years, this was only possible when some reduction in the transmission rate was also included (Fig 3a–3c). It was only possible to eliminate disease when a linear decline in bacterial load was assumed under Models 2 and 4 with at least a 10% reduction in transmission and annual MDA for 5 years (Fig 3d and 3f); in all other structures and transmission reduction scenarios, infection re-bounded. It was not possible to reach the elimination threshold guideline at all with Model 3 (Fig 3b and 3e).

When TF baseline prevalence was 40%, under Models 2 and 4, it was possible to eliminate infection within 5 years with annual MDA and an overall transmission reduction when assuming a linear decline in bacterial load (Fig 3e and 3f), provided transmission reduction was greater than 10%. However, if an exponential decline in bacterial load was assumed (Fig 3a–3c), it was only possible to eliminate infection in Model 2 with 5 annual rounds of MDA and 50% reduction in transmission, but this was not sufficient for Model 4. Under no intervention conditions evaluated here was it possible to eliminate infection with Model 3 (Fig 3b and 3e).

Assuming TF prevalence was 20% we implemented 3 annual rounds of MDA and transmission reduction (between 0–50%) (Fig 4). Considering a linear decline in bacterial load it was possible with at least 10% transmission reduction to reduce disease prevalence below the target threshold in Model 2 and 4. However, if there was no transmission reduction disease appeared to re-emerge (Fig 4d and 4f). As with the previous prevalence levels it was not possible to achieve the target level of disease prevalence with Model 3 (Fig 4e). When assuming an exponential decline in bacterial load, with all four model structures, it was possible to reduce the prevalence of disease to the target level of less than 5%. However, without subsequent rounds of MDA, this was not maintained in Model 3 and disease re-emerged (Fig 4b). However, for Models 2 and 4, 10% reduction in transmission respectively was sufficient to ensure that disease did not re-emerge in the community (Fig 4a and 4c) and suppression below the target level was maintained.

Observing the rates of re-bound under different model structures we found that for Model 2 with modest levels of transmission reduction rapid re- emergence was observed for the 4 parameter model. This is in-part likely to be because for a given prevalence level we need a higher force of infection if immunity was high, resulting in faster rates of rebound until *β* was reduced sufficiently. For Model 3 under both parameter versions and all scenarios rapid rebound of disease was seen. This is likely to be because this structure includes 2 compartments which do not contribute to the overall force of infection. Therefore in order to obtain a fixed level of prevalence the value of *β* must be increased substantially in comparison to other model structures, thus making persistence of disease more likely under this structure. Limited evidence of re-bound was seen when evaluating Model 4 in comparison to other models this model had an overall longer duration of PCR detectable infection, therefore a lower value of *β* was needed to attain any given level of prevalence. This meant that when an intervention was applied, the lower overall force of infection resulted in a slower rate of rebound. The on average higher infectivity of individuals in the 3 parameter model in comparison to the 4 parameter model (Table S4 in S1 File) is likely to explain the slightly higher re-bound rates in the 3 parameter version of Model 4, in comparison to the 4 parameter version.

We conducted one-way univariate sensitivity analyses with 6 of the fixed parameters in the model to assess their impact on the models assessment of the feasibility of elimination (Table S6 and Table S7 in S1 File). For the 4 parameter version of Model 1 and Model 2 when TF was 40% or 20%, variation in treatment efficacy and the minimum duration of infection had the largest impact on final TF prevalence. Here, higher treatment efficacy resulted in faster infection rebound, leading to a higher final TF prevalence. For Model 2 final TF prevalence was 70% compared to 61% when treatment efficacy was increased from 85% to 100% (Table S6 in S1 File). In contrast, a 50% reduction in the minimum duration of infection resulted in fast infection re-bound resulting in a high final TF prevalence above the baseline results, until a 50% reduction in transmission was implemented (Table S6 in S1 File). Decreasing the minimum duration of disease resulted in a final higher TF prevalence when transmission reduction < 50% was implemented. For the 3 parameter version of Model 1 and Model 2 final TF prevalence decreased with increasing treatment efficacy. (Table S6 in S1 File). While reduction in the minimum duration of infection and disease episodes resulted in higher final TF prevalence levels when little or no transmission reduction was implemented. Final TF prevalence for Model 2 when transmission was reduced by 10%, was 60% when the minimum duration of infection was 5 weeks, but 8% for the 10 week baseline value (Table S6 in S1 File).

Across the 3 and 4 parameter versions of Model 3, little to no variation in the final TF prevalence level was seen when sensitivity to the fixed parameters was conducted (Table S6 and Table S7 in S1 File), and the inability to even come close to reducing or eliminating disease was seen across all parameter sets tested. Insensitivity of Model 3 to perturbations in the six different parameter sets, is likely to be a consequence of the high force of infection needed to obtain a fixed level of prevalence with this model structure (as described in the model fitting results), which increases the persistence of infection and disease. Equally, individuals spend a long time in the TF +ve only state under this structure, therefore changes in infection rate parameters are less likely to have a profound impact on this model.

For Model 4, lower treatment efficacy resulted in a higher final TF prevalence than when the baseline value was used. Prevalence was 11% instead of 2% when treatment efficacy was 65% in the 4 parameter model when no transmission reduction was modelled (Table S6 in S1 File). As with Models 1 and 2, reductions in the maximum duration of infection and disease episodes at low levels of transmission reduction resulted in higher final TF prevalence for the 4 parameter model. If the maximum rate of recovery from infection was changed to 0.008 from 0.006 final TF prevalence was 40%, in comparison to 2% (Table S6 in S1 File). However, when endemic prevalence of TF was 20% for the 4 parameter model, results were consistent across all variation in the parameter sets. For the 3 parameter versions of Model 4 results were consistent across all parameters sets tested when endemic prevalence of TF was 40%. However, when endemic TF prevalence was 20% for the 3 parameter model, an increase in final TF prevalence from the baseline was observed when the minimum duration of infection and disease episodes was reduction, particularly when no transmission reduction was implemented. Final TF prevalence was 14% instead of 4% when the minimum duration of infection was decreased from 10 weeks to 5 weeks (Table S7 in S1 File).

## Discussion

In this study we present the first attempt to fit a mechanistic epidemiological model of trachoma transmission to bacterial load data, PCR and TF prevalence data across 3 different age groups. We demonstrate that it is possible to fit to the age-structured PCR data well but the very high level of TF in the youngest age group analysed in this hyper-endemic setting proved challenging to capture with all model structures tested. In addition, we highlight that predictions about the future prevalence of TF within a community can depend on the model structure assumed.

While a range of different model structures can describe the natural history of trachoma infection well, Model 2, with re-infection in the *D* state (TF positive, PCR negative), was statistically the best performing model under all conditions. Model 2 represented the most parsimonious model structure when assessed by the DIC score obtained through fitting to the dataset used here. The appropriate level of susceptibility to re-infection was, however, challenging to identify given the amount and kind of data available. We can only therefore confidently say that our model selection study suggests that individuals with active disease but no current infection remain susceptible to infection, but it does not suggest what their susceptibility to infection is, relative to those with neither active disease nor infection.

We demonstrate that overall a better fit to the data, i.e. ensuring infection and disease age-specific prevalence were captured, was provided by an exponential reduction in bacterial load in comparison to a linear decline in load. However, the use of an exponential rather than linear bacterial load decline was also shown to suggest that more effort may be required to reduce the prevalence of TF in the long term, due to the persistently high levels of load associated with those who have experienced very few prior infections i.e. those likely to be the few remaining infected individuals when elimination is close.

Estimates of the effort required and feasibility of elimination were markedly different under different model structures. Model 3 showed that the prospect of reaching TF less than 5% was very low, while with Model 2 annual MDA and transmission reduction together, in prevalence settings below 40% TF, ensured that the goal was reached within 5 years. To gain further understanding into the long-term transmission dynamics of trachoma and generate accurate elimination timelines, further insight into the duration of infection and disease episodes is required, ideally through at least one further study designed to measure these durations. Furthermore, our results highlight the importance of identifying and understanding the most appropriate and parsimonious structure to model trachoma transmission, this is essential if we wish to use mathematical models to help understand the transmission dynamics of trachoma and to model current and alternative intervention strategies.

Our sensitivity analysis highlights that projections on the feasibility of elimination under different model structures were sensitive to a number of key parameters, particularly for Models 2 and 4. Final TF prevalence after 7 years was most impacted by the assumed duration of an individual’s first infection and disease episodes, in addition to the efficacy of treatment. Suggesting that a more thorough understanding of these parameters would be valuable for future model forecasting. A small amount of variation in the final TF prevalence was observed when the maximum rates of recovery from infection and disease were perturbed, however the impact was not as profound as the outcomes from the aforementioned parameters. In general, we observed that as the modelled reduction in the transmission rate increased sensitivity of the model prediction of the final TF prevalence level decreased. However, the final TF prevalence outcomes from Model 3 appeared insensitive to perturbations across all parameter evaluated.

We were consistently unable to capture the high prevalence of TF in the youngest age group, but were able to capture PCR prevalence for this age group. This suggests that the models evaluated may be missing or mis-specifying a key component of the epidemiological system. For example, it could be that the functional form used to describe the development of immunity has been mis-specified here (as an exponential function) or that age-specific prevalence ratios of PCR vs TF vary according to the transmission setting. However, particularly for Model 2, prevalence of disease and infection were matched well for the two older age groups. However, it has been suggested that at the population level the relationship between TF and PCR positivity is approximately linear [38], which can also be seen in our model projections. Since, we have only fitted to cross-sectional data from a single time-point from a single region and time point, we cannot disregard the possibility that there may be an anomaly in the data, and extrapolating our findings to a wider context should only be done with caution. Equally, the prevalence of infection within the adult age group may be considered high. However, in the absence of cross-sectional data collected across a wide range of age groups from different study sites, it is difficult to assess whether or not this observed infection prevalence is abnormally high or not.

The models evaluated here have only been fitted to one high prevalence site, however trachoma transmission can be highly heterogeneous between neighbouring communities. Therefore it is possible that if we had used data from a different community we may have achieved different results. However our hope from fitting to data from this study site is that while certainly the baseline level of transmission within other neighbourhoods may not be as high, we would hope that some of our findings would be generalisable to other settings. Nevertheless the use of data from a single high prevalence study site is a clear limitation of the study.

Statistical models have been shown to forecast prevalence of infection and disease well and can predict changes in prevalence over time [7, 9, 12], however there is less flexibility within a statistical framework to explore the impact of novel or future alternative intervention strategies. Therefore, selecting an appropriate mechanistic model structure is important if we wish to more accurately model trachoma transmission and assess the possible impact of different intervention strategies in the lead up to 2020. Furthermore, we demonstrate that our understanding on the feasibility of trachoma elimination varies under different model structures.

In this study certainty about the appropriate model structure and susceptibility level to re-infection was hampered by a limited amount of data relating to the durations spent in different infection and disease states, in addition to longitudinal post-intervention follow-up data on infection and disease from a range of different communities and transmission settings. For example, if we knew the average duration an individual spends as PCR-positive but TF-negative we could parameterise our models with more certainty, a point that is even more important for the duration in which individuals remain only TF positive. However, in all of our models, PCR and TF positivity are inherently linked. We suggest that further validation of appropriate model structures can be provided through fitting different structures and different model types to longitudinal data from a range of different transmission settings, coupled with more large scale model and data comparisons, as we seek to develop models which help provide guidelines on time to elimination. Our findings may also be applicable to other NTDs where certain key parameters are not well known, where limited data exists and limited investigation has been done to validate the model structure being used to model transmission.

## Supporting information

S1 File Supplementary modelling information and model fits. (PDF)

S1 Data Prevalence and bacterial load data used to fit the model. (CSV)
