# Supplementary material for: Improving our forecasts for trachoma elimination: What else do we need to know?
Source: PLoS Negl Trop Dis. 2017 Feb 9;11(2):e0005378. doi: 10.1371/journal.pntd.0005378 (PMC5321453; doi:10.1371/journal.pntd.0005378)
Supplement: S1 File — (PDF) [file pntd.0005378.s001.pdf]

Supplementary information for the manuscript. Pinsent.

Mathematical models of trachoma transmission

Schematics of the four model structures evaluated are presented in Figure 1. Parameter and state variable definitions are provided in Table S1.

Detail on the structure and flow of each model has been provided in the main text, for all equations i denotes an individual's ith infection. Model equations for Model 1 and 2 are as follows:

$$\frac{\partial S_i}{\partial t}\frac{\partial a_i}{\partial t} = \int_{a'} -\lambda_a S_i(a,t) - \mu S_i(a,t) + \rho_{i+1} D_{i+1}(a,t) \tag{1}$$

$$\frac{\partial I_i}{\partial t}\frac{\partial a_i}{\partial t} = \int_{a'} \lambda_a S_i(a,t) - \mu I_i(a,t) - \sigma I_i(a,t) \tag{2}$$

$$\frac{\partial ID_i}{\partial t}\frac{\partial a_i}{\partial t} = \sigma I_i(a,t) - \mu ID_i(a,t) - \omega_i ID_i(a,t) + \int_{a'} (\lambda_a D_i(a,t))\Gamma \tag{3}$$

$$\frac{\partial D_i}{\partial t}\frac{\partial a_i}{\partial t} = \omega_i ID_i(a,t) - \mu D_i(a,t) - \rho_i D_i(a,t) - \int_{a'} (\lambda_a D_i(a,t))\Gamma \tag{4}$$

Where in Model 1 $\Gamma = 0$, and for Model 2 $\Gamma = 0.2$, 0.5 or 0.8.

The equations for Model 3 [1] are as follows:

$$\frac{\partial S_i}{\partial t}\frac{\partial a_i}{\partial t} = \int_{a'} -\lambda_a S_i(a,t) - \mu S_i(a,t) + \rho_{i+1} P D_{i+1}(a,t) \tag{5}$$

$$\frac{\partial I_i}{\partial t}\frac{\partial a_i}{\partial t} = \int_{a'} \lambda_a S_i(a,t) - \mu I_i(a,t) - \sigma I_i(a,t) \tag{6}$$

$$\frac{\partial ID_i}{\partial t}\frac{\partial a_i}{\partial t} = \sigma I_i(a,t) - \mu ID_i(a,t) - \omega_i ID_i(a,t) + \int_{a'} (\lambda_a PD_i(a,t))\Gamma \tag{7}$$

$$\frac{\partial D_i}{\partial t}\frac{\partial a_i}{\partial t} = \omega_i ID_i(a,t) - \mu D_i(a,t) - \zeta_i D_i(a,t) \tag{8}$$

$$\frac{\partial PD_i}{\partial t}\frac{\partial a_i}{\partial t} = \zeta_i D_i(a,t) - \mu PD_i(a,t) - \rho_i PD_i(a,t) - \int_{a'} (\lambda_a PD_i(a,t))\Gamma \tag{9}$$

The equations for Model 4 are as follows:

$$\frac{\partial S_i}{\partial t}\frac{\partial a_i}{\partial t} = \int_{a'} -\lambda_a S_i(a,t) - \mu S_i(a,t) + \rho_{i+1} D_{i+1}(a,t) \tag{10}$$

$$\frac{\partial I_i}{\partial t}\frac{\partial a_i}{\partial t} = \int_{a'} \lambda_a S_i(a,t) - \mu I_i(a,t) - \sigma I_i(a,t) \tag{11}$$

$$\frac{\partial IO_i}{\partial t}\frac{\partial a_i}{\partial t} = \sigma I_i(a,t) - \mu ID_i(a,t) - \eta_i ID_i(a,t) \tag{12}$$

$$\frac{\partial ID_i}{\partial t}\frac{\partial a_i}{\partial t} = \eta_i I_i(a,t) - \mu ID_i(a,t) - \omega_i ID_i(a,t) + \int_{a'} (\lambda_a D_i(a,t))\Gamma \tag{13}$$

$$\frac{\partial D_i}{\partial t}\frac{\partial a_i}{\partial t} = \omega_i ID_i(a,t) - \mu D_i(a,t) - \rho_i D_i(a,t) - \int_{a'} (\lambda_a D_i(a,t))\Gamma \tag{14}$$

For all models evaluated the force of infection was structured in the manner, therefore λ is defined as:

$$\lambda = w(a,a')\Big(\beta\Big(\big(\int_{a'} \frac{\sum_j \alpha_i ID_i(a',t)}{\sum_i N_i(a',t)}\big) + \big(\upsilon_2\big(\int_{a'} \frac{\sum_i \alpha_i ID_i(a',t)}{\sum_i N_i(a',t)}\big)^{\psi} + 1\big)\Big)\Big) \tag{15}$$

It has recently been suggested that the dynamics of trachoma may facilitate elimina-

tion [2]. Here when we implement the different interventions, we assume that individuals are exposed to a linear $\left(\int_{a'} \frac{\sum_j \alpha_i ID_i(a',t)}{\sum_i N_i(a',t)}\right)$ and non-linear $\left(v_2(\int_{a'} \frac{\sum_i \alpha_i ID_i(a',t)}{\sum_i N_i(a',t)})^{\psi} + 1\right)$ force of infection (as previously modelled). For the non-linear term, at low prevalence it behaves similarly to a mass-action model , but at mid-range prevalence levels transmission will exhibit either positive or negative feedback depending on the value of v_2 [2]. Typically in this system positive feedback allows for two stable equilibria in a deterministic model, one with zero prevalence and one with a non-zero prevalence.

α_i is the infectivity of an individual in compartment ID_i. σ is the rate at which infected individuals I_i progress to becoming infected and infectious, ω_i is the rate at which ID_i individuals recover from infection, ρ_i is the rate at which D_i individuals recover from active disease only. For Model 4 we assume that both IO and ID individuals contribute to the FOI so both are in the numerator for equation 15.

$$w(a,a') = \epsilon\delta_{a,a'} + (1-\epsilon)\frac{N_{a'}}{\sum_{a'} N_{a'}} \tag{16}$$

$w(a,a')$ is a mixing matrix which contains information on the rate of mixing between individuals of age group a and a' [3]. $\delta_(a,a')$ is the Kronecker Delta. Kronecker Delta is a piecewise function of variables, if the two variables (in this instance age-group) are equal the value of the function is 1, otherwise it is 0. Na is the number of individuals of age a' in the population and ϵ indicates the degree of mixing assortativity, which can range between 0 (random age mixing) and 1 (fully assortative).

Rate of recovery

Rates of recovery and infectivity were modelled as previously described by Gambhir et al [4]

The per individual rate of recovery ρ_i from an individuals ith infection (measured as a rate per year). The rate of recovery is assumed to change as an exponential function of i

that begins at a rate of ρ_1 (recovery from the first infection) and rises to a maximum rate ρ_100 where no greater rate of recovery can be achieved after this point [5].

$$\omega_i = \omega_1 - \omega_{100} exp[-\phi(i-1)] + \omega_{100} \tag{17}$$

We also model the per individual rate of recovery from active disease only ω_i (measured as a rate per year) from infection i the rate of recovery is assumed to change as an exponential function of i that begins at a rate of ω_1 (recovery from the first infection) and rises to a maximum rate ω_{100}. We assume the rate of change of the recovery rate per infection (ϕ) and per each disease episode (θ) were different for recovery from infection and active disease.

$$\rho_i = \rho_1 - \rho_{100} exp[-\theta(i-1)] + \rho_{100} \tag{18}$$

For the IO and PD states, we assume immunity follows the same functional form as above. We assumed the minimum duration in IO was the same as ID, and the maximum duration was half of the duration of time spent infectious estimated in [5]

Values of these parameters are provided in Table S1.

Infectivity

We assumed the infectivity of an individual α_i was proportional to their bacterial load. In the model we reflect declines in bacterial load with repeated infection as reductions in the probability of transmission. We assumed that an individuals load (i.e probability of transmitting to others in the community) decreased with an increasing number of infections experienced. Therefore, infectivity was a function of the number of previous infections experienced by each individual, a trend that is in agreement with the data from trachoma endemic communities in which the bacterial load decreases with age [6–8]. We assumed a linear decline in infectivity for the 3 parameter models, and an exponential decline in

infectivity for the 4 parameter models. These functions also saturated after 100 infections have been experienced. For all 4 of the different model structures evaluated in the analysis we fit them as both 3 and 4 parameter models. For all 4 of the different model structures evaluated in the analysis we fit each of them as both 3 and 4 parameter models.

The rate of decline in infectivity for the exponential decline in infectivity was:

$$\alpha_i = \alpha_1 - \alpha_{100} exp[-\pi(i-1)] + \alpha_{100} \quad (19)$$

Model parameters and state variables

Table S1 provides definitions of all state variables, parameter definitions and values.

Model fitting

All parameter estimates in the 3 and 4 parameter model (β, α_1, ρ_1, ω_1) (Table S1) were performed using Markov Chain Monte Carlo (MCMC). Each MCMC chain was run for 10,000 iterations, The Robbins-Munro algorithm was implemented as part of the adaptive stage of the MCMC-Metropolis Hastings algorithm, to ensure the proposal distributions were adaptively tuned ensuring efficient exploration of the posterior [11]. Median posterior density provided the point estimate of the parameters. Credible intervals for each parameter estimate for each model were derived as the 2.5 th and 97.5 th percentiles of the posterior density.

For each parameter set we calculate the log-likelihood (LL), as previously done [4] of observing the age-specific data on prevalence of infection and disease, as well as the rates of recovery and infection load.

Table S1: State variables, parameters definitions and values used in the model. Where two numbers are listed for ψ, they indicate the values used for TF 40% and 20% communities.

Name	Definition	Value	Units	Source
S_i	Susceptible individuals		Number	
I_i	Infected but not infectious		Number	
ID_i	Infected and Infectious (PCR and TF +ve)		Number	
D_i	Diseased and not infectious (TF +ve)		Number	
PD_i	Partially diseased can be re-infected (TF +ve)		Number	
IO_i	Infected and infectious (PCR +ve)		Number	
β	Transmission rate parameter	Estimated	Proportion	
ϵ	Degree of random mixing in the population	0.5	Proportion	[4]
c	Coverage level of treatment	80%	Percentage	
e	Efficacy of treatment	85%	Percentage	[9, 10]
N_{infs}	Maximum number of infections before immunity saturates	100	Number	[4]
N	Total number of indivduals in the population	6000	Number	
α	Infectivity of an individual proportional to the log of their bacterial load	0 - 1	Proportion	[4]
ρ_1	Minimum rate of recovery from active disease after 1st infection	Estimated	Day^{-1}	[4, 5]
ρ_{100}	Maximum rate of recovery from active disease after 100th infection	1/7	Day^{-1}	[4, 5]
ω_1	Minimum rate of recovery from 1st infection	Estimated	Day^{-1}	[4, 5]
ω_{100}	Maximum rate of recovery from 100th infection	1/77	Day^{-1}	[4, 5]
θ	Rate of change of the recovery from disease rate per infection	0.30	Proportion	[4, 5]
ϕ	Rate of change of the recovery from infection rate per infection	0.45	Proportion	[5]
π	Rate of change of infectivity rate per infection	Estimated	Proportion	[4, 5]
ψ	Non-linear power term	1.2, 1.4	Number	[2]
ζ_1	Rate of recovery from PD with 1st infection	1/134	Day^{-1}	[1, 5]
ζ_{100}	Rate of recovery from PD with 100th infection	1/7	Day^{-1}	[1, 5]
η_1	Rate of recovery from IO with 1st infection	1/77	Day^{-1}	[5]
η_{100}	Rate of recovery from IO with 100th infection	1/38	Day^{-1}	[5]
σ	Rate at which infected individuals become infectious	1/14	Day^{-1}	[5]
λ_a	Age-specific force of infection	Calculated		
υ_2	Non-linear constant term	2.6	Number	[2]
Γ	Susceptibility to re-infection in the disease state	0, 0.20, 0.50, 0.80	Proportion	[1]

We assumed a binomial likelihood for the prevalence data on infection and disease.

$$LL_{prev} = \sum_{a} lnp_a + (n_a - x_a)ln(1 - p_a) \tag{20}$$

where p_a is the model-predicted infection or disease prevalence for age group a; x_a is the number of individuals that test positive for infection or disease and n_a the total number of individuals in the data set for age group a.

To estimate the rate of recovery from an individuals first infection and disease episode as well as the rate of change in infectivity (as determined by infection load) we evaluated a Gaussian likelihood for each parameter separately.

$$LL_{duration and load} = \sum_{a} \frac{-ln\sqrt{Var_a} - (D_a - M_a^2)}{2Var_a} \tag{21}$$

Where Var_a is the age-specific fixed variance. Variance of the bacterial load and infection and disease durations for each age group was fixed due to the issue arising that some parameter sets that resulted in poorer fits to the data but minimised the variance resulted in better performing likelihoods. Therefore it was chosen to keep the variance constant across all parameter sets. The variance was fixed when the 3 and 4 parameter models were fit and for the iterations of the MCMC chain within each model. Variance was 128, 98, 32 days for each age group respectively for the duration of an infection and disease episode. Higher variance was assumed for the durations of infection and disease for the younger age group reflecting large uncertainty in previous estimates of these parameters [5]. For bacterial load a variance of 0.005 was used across all age groups. D_a is the value in the data and M_a is the model predicted value for a given set of parameters.

Therefore the 3 and 4 parameter models, the likelihood of each parameter set was eval-

uated as the sum of each of the individual likelihood for each parameter.

$$LL_{total} = LL_{previnfection} + LL_{prevdisease} + LL_{infectionrecover} + LL_{diseaserecover} + LL_{loadrecover} \quad (22)$$

Modelling interventions

In the analysis two interventions were modelled. Firstly we modelled the implementation of Facial cleanliness and Environmental improvements (F and E) as a reduction in the transmission rate parameter β. We considered 0, 10, 30 and 50% reductions in the transmission rate parameter over the intervention period.

We also performed annual rounds of mass drug administration. The total number of people effectively treated in each annual round was calculated as $c * e$, and this treatment was performed for all individuals who were in a PCR positive state. Treatment was assumed to have no impact on an individuals disease state. Therefore, members of the population that were treated successfully in the I_i state were returned to the S_i, thus we assumed that no improved immunity developed as a result of this infection. Individuals that were in the ID or IO states who cleared their infection following treatment progressed to the D state, hence they non-longer had a PCR detectable infection but were still TF positive. We assumed that immunity to infection did develop if an individual was treated in the ID or IO states. A schematic of this movement due to treatment is illustrated in Figure 1.

Results: Model fits and parameter estimates

Age specific fits to the bacterial load data are presented in Figure S1 and Table S4.

MCMC diagnostics to assess convergence of 2 MCMC chains using the Gelman-Rubin statistic (R_c) and the Effective Sample Size (ESS) of each posterior (Table S5). Ensuring

Table S2: **Parameter estimates from the 10 four parameter models.** Estimates of the transmission rate parameter β day^{-1}, the rate of reduction in infectivity with each sucessive infection, the rate of recovery from an individual's first infection and the rate of recovery from an individual's first disease episode. Numbers in brackets to the right of each point estimate indicate the 95% credible intervals associated with that estimate.

Model number	β day^{-1}	bacload	IA max	D max	DIC
**Model 1**					
0% susceptibility	0.083 (0.068 - 0.010)	0.108 (0.081 - 0.144)	0.0042 (0.0028 - 0.007)	0.0031 (0.0028 - 0.0034)	1942.44
**Model 2**					
20% susceptibility	0.072 (0.058 - 0.089)	0.108 (0.078 - 0.145)	0.0047 (0.0036 - 0.0067)	0.0032 (0.0029 - 0.0035)	1931.18
50% susceptibility	0.061 (0.050 - 0.083)	0.114 (0.083 - 0.190)	0.005 (0.004 - 0.008)	0.0033 (0.0030 - 0.0037)	1930.45
80% susceptibility	0.053 (0.045 - 0.064)	0.118 (0.084 - 0.168)	0.006 (0.005 - 0.008)	0.0035 (0.0032 - 0.0038)	1928.04
**Model 3**					
20% susceptibility	0.690 (0.520 - 0.920)	0.180 (0.130 - 0.244)	0.0045 (0.0039 - 0.0053)	0.0037 (0.0035 - 0.0040)	1949.79
50% susceptibility	0.601 (0.459 - 0.824)	0.178 (0.127 - 0.256)	0.0049 (0.0043 - 0.0057)	0.0041 (0.0038 - 0.0044)	1951.82
80% susceptibility	0.560 (0.436 - 0.750)	0.190 (0.138 - 0.267)	0.0052 (0.0046 - 0.0062)	0.0043 (0.0041 - 0.0046)	1954.81
**Model 4**					
20% susceptibility	0.036 (0.030 - 0.049)	0.443 (0.314 - 0.587)	0.0064 (0.0055 - 0.0082)	0.0013 (0.0078 - 0.189)	1961.97
50% susceptibility	0.035 (0.027 - 0.049)	0.459 (0.309 - 0.631)	0.0006 (0.0005 - 0.0008)	0.136 (0.085 - 0.241)	1956.65
80% susceptibility	0.036 (0.031 - 0.091)	0.463 (0.345 - 0.592)	0.0006 (0.0005 - 0.0008)	0.145 (0.093 - 0.180)	1950.60

Table S3: **Estimates from the 10 three parameter models.** Estimates of the transmission rate parameter β day^{-1}, the rate of reduction in infectivity with each sucessive infection, the rate of recovery from an individual's first infection and the rate of recovery from an individual's first disease episode. Numbers in brackets to the right of each point estimate indicate the 95% credible intervals associated with that estimate.

Model number	β	IA max	D max	DIC
**Model 1**				
0% susceptibility	0.030 (0.027 - 0.032)	0.0043 (0.0031 - 0.0068)	0.0029 (0.0026 - 0.0032)	2091.78
**Model 2**				
80% susceptibility	0.028 (0.027 - 0.030)	0.0048 (0.0035 - 0.0068)	0.0029 (0.0027 - 0.0032)	2071.27
50% susceptibility	0.026 (0.025 - 0.028)	0.0053 (0.0040 - 0.0076)	0.0030 (0.0027 - 0.0033)	2058.53
80% susceptibility	0.024 (0.023 - 0.026)	0.0063 (0.0046 - 0.0092)	0.0031 (0.0028 - 0.0034)	2033.58
**Model 3**				
20% susceptibility	0.179 (0.159 - 0.201)	0.0044 (0.0038 - 0.0040)	0.0038 (0.0035 - 0.0040)	2145.64
50% susceptibility	0.167 (0.150 - 0.187)	0.0047 (0.0041 - 0.0054)	0.0040 (0.0038 - 0.0043)	2145.37
80% susceptibility	0.156 (0.141 - 0.173)	0.0049 (0.0044 - 0.0058)	0.0043 (0.0040 - 0.0045)	2173.60
**Model 4**				
20% susceptibility	0.036 (0.033 - 0.040)	0.0038 (0.0035 - 0.0044)	0.0033 (0.0031 - 0.0035)	2484.80
50% susceptibility	0.032 (0.029 - 0.035)	0.0044 (0.0040 - 0.0049)	0.0033 (0.0032 - 0.0036)	2452.25
80% susceptibility	0.030 (0.027 - 0.033)	0.0049 (0.0044 - 0.0056)	0.0034 (0.0032 - 0.0037)	2443.66

Table S4: Age specific fits to the bacterial load data, each number represents age groups 1, 2 and 3 respectively.

Model number	Load
**Data**	0.80, 0.30, 0.10
**Model 1 - 4 parameters**	0.54, 0.26, 0.08
**Model 2 - 4 parameters**	
20% susceptibility	0.58, 0.29 , 0.08
50% susceptibility	0.62, 0.31, 0.09
80% susceptibility	0.64, 0.34, 0.10
**Model 3 - 4 parameters**	
20% susceptibility	0.53, 0.22, 0.05
50% susceptibility	0.55, 0.25, 0.06
80% susceptibility	0.56, 0.25, 0.05
**Model 4 - 4 parameters**	
20% susceptibility	0.63, 0.46, 0.19
50% susceptibility	0.61, 0.45, 0.20
80% susceptibility	0.61, 0.45, 0.19
**Model 1 - 3 parameters**	
0% susceptibility	0.94, 0.82, 0.49
**Model 2 - 3 parameters**	
20% susceptibility	0.94, 0.82, 0.49
50% susceptibility	0.95, 0.84, 0.52
80% susceptibility	0.95, 0.85, 0.54
**Model 3 - 3 parameters**	
20% susceptibility	0.96, 0.86, 0.59
50% susceptibility	0.96, 0.86, 0.60
80% susceptibility	0.97, 0.87, 0.61
**Model 4 - 3 parameters**	
20% susceptibility	0.99, 0.96, 0.87
50% susceptibility	0.99, 0.99, 0.93
80% susceptibility	0.99, 0.99, 0.96

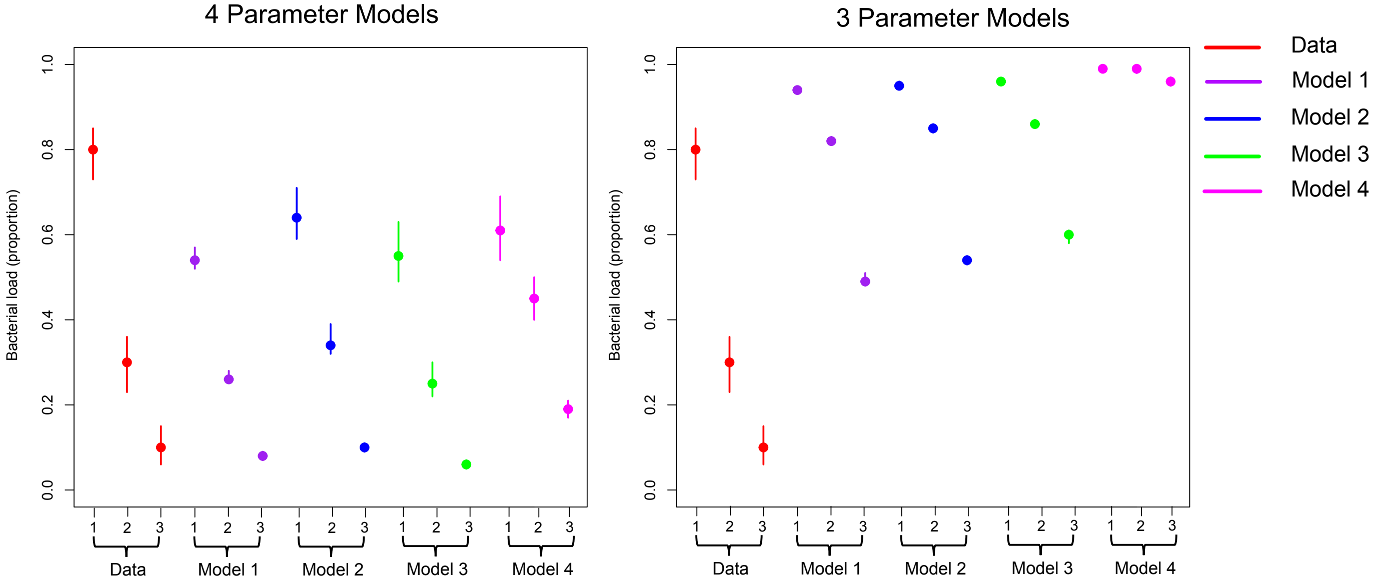

Figure S1: **Estimates from the best performing models of the age-specific bacterial load data.** Estimates of age-specific bacterial load data from statistically the best performing models for each structure evaluated. Data is shown in red, Model 1 results are shown in purple, Model 2 results are shown in blue, Model 3 results are shown in green, and Model 4 results are shown in pink. The left figure shows fits from the 4 parameter models. The right figure shows fits from the 3 parameter model. Lines around each models point estimate are the 95% credible intervals.

that $R_c < 1.1$ and that the ESS was > 150.

Sensitivity of the model predictions to fixed parameters

Table S5: MCMC diagnostics for each model fit, we present the Gelman-Rubin statistic for each parameter estimated and the value of the smallest value of the effective sample for the parameters estimated.

Model number	Gelman-Rubin statistic	Effective Sample Size
**Model 1 - 4 parameters**	1.02, 1.02, 1.00, 1.00	> 200
**Model 2 - 4 parameters**		
20% susceptibility	1.02, 1.02, 1.00, 1.00	> 150
50% susceptibility	1.04, 1.04, 1.00, 1.00	> 150
80% susceptibility	1.00, 1.00, 1.01, 1.00	> 150
**Model 3 - 4 parameters**		
20% susceptibility	1.00, 1.00, 1.00, 1.00	> 200
50% susceptibility	1.00, 1.00, 1.00, 1.00	> 200
80% susceptibility	1.00, 1.00, 1.00, 1.00	> 200
**Model 4 - 4 parameters**		
20% susceptibility	1.10, 1.10, 1.10, 1.10	> 150
50% susceptibility	1.10, 1.00, 1.05, 1.10	> 150
80% susceptibility	1.09, 1.01, 1.02, 1.09	> 150
**Model 1 - 3 parameters**		
0% susceptibility	1.00, 1.00, 1.00	> 200
**Model 2 - 3 parameters**		
20% susceptibility	1.00, 1.03, 1.00	> 150
50% susceptibility	1.00, 1.00, 1.00	> 200
80% susceptibility	1.00, 1.08, 1.00	> 150
**Model 3 - 3 parameters**		
20% susceptibility	1.00, 1.01, 1.00	> 150
50% susceptibility	1.00, 1.03, 1.00	> 150
80% susceptibility	1.00, 1.00, 1.02	> 150
**Model 4 - 3 parameters**		
20% susceptibility	1.00, 1.00, 1.00	> 200
50% susceptibility	1.00, 1.00, 1.00	> 200
80% susceptibility	1.00, 1.00, 1.00	> 200

Table S6: For each model number with the lowest DIC score we assess the sensitivity of the model predictions to fixed parameters for the TF 40% setting. We varied parameters that were fixed but not estimated in the model by +/- 50% of the assumed baseline value. For those parameters that were estimated we used the upper and lower values from the 95% credible intervals, these are indicated with a *.

Model number and varied param	Parameter values evaluated	Final TF prevalence (0% F& E)	Final TF prevalence (10% F& E)	Final TF prevalence (30% F& E)	Final TF prevalence (50% F& E)
**Model 1 - 4 parameters - 0% susceptibility**					
Treatment efficacy	0.65, 0.85, 1	0.47, 0.49, 0.56	0.46, 0.50, 0.60	0.40, 0.44, 0.25	0.08, 0.04, 0.00
Duration of 1st infection	5, 10, 15	0.40, 0.49, 0.51	0.39,0.50,0.52	0.36, 0.44, 0.41	0.36, 0.04, 0.03
Duration of 1st disease episode	0.5, 1, 2	0.44, 0.49, 0.44	0.43, 0.50, 0.43	0.44, 0.44, 0.44	0.27, 0.04, 0.27
Maximum rate of infection recovery*	0.0028, 0.004, 0.007	0.47, 0.49, 0.42	0.48, 0.50, 0.40	0.51, 0.44, 0.38	0.17, 0.04, 0.32
Maximum rate of disease recovery*	0.0028, 0.0031, 0.0034	0.44, 0.49, 0.44	0.43, 0.50, 0.44	0.44, 0.44, 0.44	0.27, 0.04, 0.35
Degree of age-group mixing	0.25, 0.50, 0.75	0.43, 0.49, 0.49	0.43, 0.50, 0.51	0.43, 0.44, 0.36	0.35, 0.04, 0.02
**Model 2 - 4 parameters - 80% susceptibility**					
Treatment efficacy	0.65, 0.85, 1	0.53, 0.61, 0.70	0.51, 0.60, 0.33	0.11, 0.06, 0.00	0.00, 0.00, 0.00
Duration of 1st infection	5, 10, 15	0.48, 0.61, 0.34	0.49,0.60,0.09	0.52, 0.06, 0.00	0.00, 0.00, 0.00
Duration of 1st disease episode	0.5, 1, 2	0.57, 0.61, 0.66	0.59, 0.60, 0.47	0.11, 0.06, 0.05	0.08, 0.00, 0.02
Maximum rate of infection recovery*	0.005, 0.006, 0.008	0.64, 0.61, 0.61	0.53, 0.60, 0.60	0.04, 0.06, 0.05	0.00, 0.00, 0.00
Maximum rate of disease recovery*	0.0032, 0.0035, 0.0038	0.62, 0.61, 0.61	0.59, 0.60, 0.61	0.04, 0.06, 0.08	0.00, 0.00, 0.00
Degree of age-group mixing	0.25, 0.50, 0.75	0.60, 0.61, 0.62	0.61, 0.60, 0.56	0.09, 0.06, 0.04	0.00, 0.00, 0.00
**Model 3 - 4 parameters - 20% susceptibility**					
Treatment efficacy	0.65, 0.85, 1	0.40, 0.40, 0.40	0.38, 0.38, 0.38	0.35, 0.35, 0.35	0.30, 0.30, 0.30
Duration of 1st infection	5, 10, 15	0.40, 0.40, 0.40	0.38, 0.38, 0.38	0.35, 0.35, 0.35	0.31, 0.30, 0.30
Duration of 1st disease episode	0.5, 1, 2	0.40, 0.40, 0.40	0.37, 0.38, 0.38	0.34, 0.35, 0.35	0.30, 0.30, 0.30
Maximum rate of infection recovery*	0.0039, 0.0045, 0.0053	0.40, 0.40, 0.40	0.39, 0.38, 0.37	0.35, 0.35, 0.33	0.31, 0.30, 0.28
Maximum rate of disease recovery*	0.0035, 0.0037, 0.0040	0.40, 0.40, 0.40	0.38, 0.38, 0.38	0.35, 0.35, 0.35	0.30, 0.30, 0.30
Degree of age-group mixing	0.25, 0.50, 0.75	0.40, 0.40, 0.40	0.38, 0.38, 0.38	0.35, 0.35, 0.35	0.30, 0.30, 0.30
**Model 4 - 4 parameters - 80% susceptibility**					
Treatment efficacy	0.65, 0.85, 1	0.11, 0.02, 0.00	0.08, 0.01, 0.00	0.03, 0.00, 0.00	0.01, 0.00, 0.00
Duration of 1st infection	5, 10, 15	0.07, 0.02, 0.01	0.04, 0.01, 0.00	0.00, 0.00, 0.00	0.00, 0.00, 0.00
Duration of 1st disease episode	0.5, 1, 2	0.02, 0.02, 0.02	0.01, 0.01, 0.01	0.00, 0.00, 0.00	0.00, 0.00, 0.00
Maximum rate of infection recovery*	0.005, 0.006, 0.008	0.02, 0.02, 0.40	0.01, 0.01, 0.26	0.00, 0.00, 0.06	0.00, 0.00, 0.01
Maximum rate of disease recovery*	0.093, 0.145, 0.180	0.05, 0.02, 0.07	0.03, 0.01, 0.03	0.01, 0.00, 0.01	0.00, 0.00, 0.00
Degree of age-group mixing	0.25, 0.50, 0.75	0.02, 0.02, 0.05	0.01, 0.01, 0.03	0.00, 0.00, 0.00	0.00, 0.00, 0.00
**Model 1 - 3 parameters - 0% susceptibility**					
Treatment efficacy	0.65, 0.85, 1	0.49, 0.46, 0.47	0.42, 0.23, 0.16	0.09, 0.01, 0.00	0.00, 0.00, 0.00
Duration of 1st infection	5, 10, 15	0.44, 0.46, 0.05	0.44, 0.23, 0.00	0.49, 0.01, 0.00	0.06, 0.00, 0.00
Duration of 1st disease episode	0.5, 1, 2	0.26, 0.46, 0.43	0.07, 0.23, 0.18	0.00, 0.01, 0.01	0.00, 0.00, 0.00
Maximum rate of infection recovery*	0.0046, 0.0063, 0.0092	0.47, 0.46, 0.45	0.22, 0.23, 0.25	0.01, 0.01, 0.00	0.00, 0.00, 0.00
Maximum rate of disease recovery*	0.0028, 0.0031, 0.0034	0.46, 0.46, 0.45	0.23, 0.23, 0.22	0.03, 0.01, 0.01	0.00, 0.00, 0.00
Degree of age-group mixing	0.25, 0.50, 0.75	0.47, 0.46, 0.50	0.24, 0.23, 0.36	0.01, 0.01, 0.03	0.00, 0.00, 0.00
**Model 2 - 3 parameters - 80% susceptibility**					
Treatment efficacy	0.65, 0.85, 1	0.23, 0.08, 0.01	0.07, 0.02, 0.00	0.00, 0.00, 0.00	0.00, 0.00, 0.00
Duration of 1st infection	5, 10, 15	0.60, 0.08, 0.02	0.54, 0.02, 0.00	0.01, 0.00, 0.00	0.00, 0.00, 0.00
Duration of 1st disease episode	0.5, 1, 2	0.11, 0.08, 0.02	0.02, 0.02, 0.01	0.00, 0.00, 0.00	0.00, 0.00, 0.00
Maximum rate of infection recovery*	0.0046, 0.0063, 0.0092	0.07, 0.08, 0.12	0.01, 0.02, 0.02	0.00, 0.00, 0.00	0.00, 0.00, 0.00
Maximum rate of disease recovery*	0.0028, 0.0031, 0.0034	0.08, 0.08,0.11	0.02, 0.02, 0.02	0.00, 0.00, 0.00	0.00, 0.00, 0.00
Degree of age-group mixing	0.25, 0.50, 0.75	0.10, 0.08, 0.02	0.02, 0.02, 0.01	0.00, 0.00, 0.00	0.00, 0.00, 0.00
**Model 3 - 3 parameters - 50% susceptibility**					
Treatment efficacy	0.65, 0.85, 1	0.40, 0.40, 0.39	0.37, 0.37, 0.37	0.30, 0.30, 0.30	0.24, 0.24, 0.23
Duration of 1st infection	5, 10, 15	0.40, 0.40, 0.40	0.38, 0.37, 0.37	0.35, 0.30, 0.30	0.30, 0.24, 0.28
Duration of 1st disease episode	0.5, 1, 2	0.40, 0.40, 0.40	0.39, 0.37, 0.37	0.33, 0.30, 0.33	0.26, 0.24, 0.27
Maximum rate of infection recovery*	0.0041, 0.0047 , 0.0054	0.40, 0.40, 0.40	0.38, 0.37, 0.34	0.34, 0.30, 0.33	0.28, 0.24, 0.26
Maximum rate of disease recovery*	0.0038, 0.0040, 0.0043	0.40, 0.40, 0.40	0.38, 0.37, 0.37	0.34, 0.30, 0.33	0.27, 0.24, 0.26
Degree of age-group mixing	0.25, 0.50, 0.75	0.40, 0.40, 0.40	0.38, 0.37, 0.37	0.34, 0.30, 0.33	0.27, 0.24, 0.27
**Model 4 - 3 parameters - 80% susceptibility**					
Treatment efficacy	0.65, 0.85, 1	0.40, 0.41, 0.41	0.37, 0.39, 0.40	0.33, 0.38, 0.40	0.07, 0.05, 0.00
Duration of 1st disease episode	5, 10, 15	0.40, 0.41, 0.42	0.37, 0.39, 0.40	0.32, 0.38, 0.20	0.28, 0.05, 0.00
Duration of 1st disease episode	0.5, 1, 2	0.40, 0.41, 0.42	0.37, 0.39, 0.40	0.32, 0.38, 0.35	0.28, 0.05, 0.00
Maximum rate of infection recovery*	0.0044, 0.0049, 0.0056	0.40, 0.41, 0.40	0.38, 0.39, 0.38	0.39, 0.38, 0.36	0.03, 0.05, 0.05
Maximum rate of disease recovery*	0.0032, 0.0034, 0.0037	0.40, 0.41, 0.40	0.38, 0.39, 0.38	0.38, 0.38, 0.38	0.03, 0.05, 0.03
Degree of age-group mixing	0.25, 0.50, 0.75	0.40, 0.41, 0.40	0.38, 0.39, 0.38	0.36, 0.38, 0.36	0.07, 0.05, 0.03

Table S7: For each model number with the lowest DIC score we assess the sensitivity of the model predictions to fixed parameters for the TF 20% setting. We varied parameters that were fixed but not estimated in the model by +/- 50% of the assumed baseline value. For those parameters that were estimated we used the upper and lower values from the 95% credible intervals.

Model number and varied param	Parameter values evaluated	Final TF prevalence (0% F& E)	Final TF prevalence (10% F& E)	Final TF prevalence (30% F& E)	Final TF prevalence (50% F& E)
**Model 1 - 4 parameters - 0% susceptibility**					
Treatment efficacy	0.65, 0.85, 1	0.26, 0.11, 0.06	0.13, 0.07, 0.02	0.02, 0.00, 0.00	0.00, 0.00, 0.00
Duration of 1st infection	5, 10, 15	0.40, 0.11, 0.05	0.41, 0.07, 0.03	0.32, 0.00, 0.01	0.16, 0.00, 0.00
Duration of 1st disease episode	0.5, 1, 2	0.10, 0.11, 0.10	0.08, 0.07, 0.07	0.03, 0.00, 0.03	0.00, 0.00, 0.00
Maximum rate of infection recovery*	0.0028, 0.004, 0.007	0.05, 0.11, 0.33	0.03, 0.07, 0.29	0.02, 0.00, 0.12	0.00, 0.00, 0.02
Maximum rate of disease recovery*	0.0028, 0.0031, 0.0034	0.16, 0.11, 0.16	0.12, 0.07, 0.11	0.05, 0.00, 0.04	0.02, 0.00, 0.01
Degree of age-group mixing	0.25, 0.50, 0.75	0.15, 0.11, 0.06	0.10, 0.07, 0.04	0.04, 0.00, 0.02	0.01, 0.00, 0.00
**Model 2 - 4 parameters - 80% susceptibility**					
Treatment efficacy	0.65, 0.85, 1	0.29, 0.23, 0.11	0.11, 0.06, 0.02	0.00, 0.00, 0.00	0.00, 0.00, 0.00
Duration of 1st infection	5, 10, 15	0.44, 0.23, 0.10	0.36, 0.06, 0.02	0.01, 0.00, 0.00	0.00, 0.00, 0.00
Duration of 1st disease episode	0.5, 1, 2	0.31, 0.23, 0.16	0.09, 0.06, 0.04	0.11, 0.00, 0.00	0.00, 0.00, 0.00
Maximum rate of infection recovery*	0.005, 0.006, 0.0065	0.19, 0.23, 0.25	0.05, 0.06, 0.06	0.00, 0.00, 0.00,	0.00, 0.00, 0.00
Maximum rate of disease recovery*	0.0032, 0.0035, 0.0038	0.16, 0.23, 0.27	0.05, 0.06, 0.07	0.00, 0.00, 0.00	0.00, 0.00, 0.00
Degree of age-group mixing	0.25, 0.50, 0.75	0.20, 0.23, 0.21	0.06, 0.06, 0.05	0.00, 0.00, 0.00	0.00, 0.00, 0.00
**Model 3 - 4 parameters - 20% susceptibility**					
Treatment efficacy	0.65, 0.85, 1	0.20, 0.20, 0.19	0.17, 0.17, 0.17	0.14, 0.14, 0.14	0.10, 0.10, 0.10
Duration of 1st infection	5, 10, 15	0.20, 0.20, 0.18	0.18, 0.17, 0.20	0.15, 0.14, 0.13	0.11, 0.10, 0.10
Duration of 1st disease episode	0.5, 1, 2	0.18, 0.20, 0.20	0.16, 0.17, 0.17	0.13, 0.14, 0.14	0.10, 0.10, 0.10
Maximum rate of infection recovery*	0.0039, 0.0045, 0.0053	0.19, 0.20, 0.20	0.17, 0.17, 0.17	0.14,0.14, 0.14	0.10, 0.10, 0.10
Maximum rate of disease recovery*	0.0035, 0.0037, 0.0040	0.20, 0.20, 0.19	0.18, 0.17, 0.17	0.14, 0.14, 0.14	0.10, 0.10, 0.10
Degree of age-group mixing	0.25, 0.50, 0.75	0.19, 0.20, 0.20	0.17, 0.17, 0.17	0.14, 0.14, 0.14	0.10, 0.10, 0.10
**Model 4 - 4 parameters - 80% susceptibility**					
Treatment efficacy	0.65, 0.85, 1	0.20, 0.20, 0.20	0.19, 0.19, 0.18	0.15, 0.15, 0.15	0.13, 0.13, 0.13
Duration of 1st infection	5, 10, 15	0.18, 0.20, 0.20	0.02, 0.00, 0.00	0.00, 0.00, 0.00	0.00, 0.00, 0.00
Duration of 1st disease episode	0.5, 1, 2	0.20, 0.20, 0.20	0.02, 0.00, 0.00	0.00, 0.00, 0.00	0.00, 0.00, 0.00
Maximum rate of infection recovery*	0.005, 0.006, 0.008	0.19, 0.20, 0.20	0.02, 0.00, 0.00	0.00, 0.00, 0.00	0.00, 0.00, 0.00
Maximum rate of disease recovery*	0.093, 0.145, 0.180	0.20, 0.20, 0.20	0.02, 0.00, 0.00	0.00, 0.00, 0.00	0.00, 0.00, 0.00
Degree of age-group mixing	0.25, 0.50, 0.75	0.18, 0.20, 0.20	0.02, 0.00, 0.00	0.00, 0.00, 0.00	0.00, 0.00, 0.00
**Model 1 - 3 parameters - 0% susceptibility**					
Treatment efficacy	0.65, 0.85, 1	0.18, 0.11, 0.04	0.13, 0.06, 0.02	0.05, 0.01, 0.00	0.01, 0.00, 0.00
Duration of 1st infection	5, 10, 15	0.32, 0.11, 0.06	0.37, 0.06, 0.03	0.31, 0.01, 0.01	0.10, 0.00, 0.00
Duration of 1st disease episode	0.5, 1, 2	0.19, 0.11, 0.12	0.13, 0.06, 0.07	0.03, 0.01, 0.02	0.00, 0.00, 0.00
Maximum rate of infection recovery*	0.0046, 0.0063, 0.0092	0.09, 0.11, 0.13	0.05, 0.06, 0.06	0.02, 0.01, 0.01	0.00, 0.00, 0.00
Maximum rate of disease recovery*	0.0028, 0.0031, 0.0034	0.11, 0.11, 0.11	0.42, 0.06, 0.06	0.09, 0.01, 0.01	0.00, 0.00, 0.00
Degree of age-group mixing	0.25, 0.50, 0.75	0.12, 0.11, 0.16	0.07, 0.06, 0.09	0.02, 0.01, 0.03	0.00, 0.00, 0.00
**Model 2 - 3 parameters - 80% susceptibility**					
Treatment efficacy	0.65, 0.85, 1	0.08 , 0.03, 0.00	0.02, 0.00, 0.00	0.00, 0.00, 0.00	0.00, 0.00, 0.00
Duration of 1st infection	5, 10, 15	0.26 , 0.03, 0.01	0.05, 0.00, 0.00	0.00, 0.00, 0.00	0.00, 0.00, 0.00
Duration of 1st disease episode	0.5, 1, 2	0.03, 0.03, 0.03	0.00, 0.00, 0.00	0.00, 0.00, 0.00	0.00, 0.00, 0.00
Maximum rate of infection recovery*	0.0046, 0.0063, 0.0092	0.03, 0.03, 0.02	0.00, 0.00, 0.00	0.00, 0.00, 0.00	0.00, 0.00, 0.00
Maximum rate of disease recovery*	0.0027, 0.0031 , 0.0034	0.01, 0.03, 0.03	0.02, 0.00, 0.00	0.00, 0.00, 0.00	0.00, 0.00, 0.00
Degree of age-group mixing	0.25, 0.50, 0.75	0.03, 0.03, 0.03	0.00, 0.00, 0.00	0.00, 0.00, 0.00	0.00, 0.00, 0.00
**Model 3 - 3 parameters - 50% susceptibility**					
Treatment efficacy	0.65, 0.85, 1	0.20, 0.20, 0.19	0.18, 0.17, 0.17	0.14, 0.13, 0.13	0.10, 0.10, 0.10
Duration of 1st infection	5, 10, 15	0.18, 0.20, 0.20	0.17, 0.17, 0.17	0.13, 0.13, 0.13	0.10, 0.10, 0.10
Duration of 1st disease episode	0.5, 1, 2	0.18, 0.20, 0.20	0.16, 0.17, 0.17	0.13, 0.13, 0.13	0.10, 0.10, 0.10
Maximum rate of infection recovery*	0.0041, 0.0047, 0.0054	0.20, 0.20, 0.18	0.17, 0.17, 0.16	0.13, 0.13, 0.12	0.10, 0.10, 0.09
Maximum rate of disease recovery*	0.0038, 0.0040, 0.0043	0.19, 0.20, 0.19	0.17, 0.17, 0.17	0.13, 0.13, 0.13	0.10, 0.10, 0.10
Degree of age-group mixing	0.25, 0.50, 0.75	0.20, 0.20, 0.18	0.18, 0.17, 0.16	0.14, 0.13, 0.13	0.10, 0.10, 0.10
**Model 4 - 3 parameters - 80% susceptibility**					
Treatment efficacy	0.65, 0.85, 1	0.11, 0.04, 0.01	0.03, 0.01, 0.00	0.00, 0.00, 0.00	0.00, 0.00, 0.00
Duration of 1st infection	5, 10, 15	0.14, 0.04, 0.03	0.04, 0.01, 0.01	0.00, 0.00, 0.00	0.00, 0.00, 0.00
Duration of 1st disease episode	0.5, 1, 2	0.07, 0.04, 0.05	0.02, 0.01, 0.01	0.00, 0.00, 0.00	0.00, 0.00, 0.00
Maximum rate of infection recovery*	0.0040, 0.0044, 0.0054	0.06, 0.04, 0.07	0.02, 0.01, 0.01	0.00, 0.00, 0.00	0.00, 0.00, 0.00
Maximum rate of disease recovery*	0.0032, 0.0033, 0.0036	0.05, 0.04, 0.01	0.02, 0.01, 0.00	0.00, 0.00, 0.00	0.00, 0.00, 0.00
Degree of age-group mixing	0.25, 0.50, 0.75	0.04, 0.04, 0.05	0.01, 0.01, 0.02	0.00, 0.00, 0.00	0.00, 0.00, 0.00

References

[1] Shattock AJ, Gambhir M, Taylor HR, Cowling CS, Kaldor JM, Wilson DP. Control of Trachoma in Australia: A Model Based Evaluation of Current Interventions. PLoS Neglected Tropical Diseases. 2015;9(4):1–12.

[2] Lietman TM, Gebre T, Ayele B, Ray KJ, Maher MC, See CW, et al. The epidemiological dynamics of infectious trachoma may facilitate elimination. Epidemics. 2011;3(2):119 – 124.

[3] Anderson RM, May RMRM. Infectious diseases of humans : dynamics and control. Oxford science publications. Oxford, New York: Oxford University Press; 1991.

[4] Gambhir M, Basanez MG, Burton MJ, Solomon AW, Bailey RL, Holland MJ, et al. The Development of an Age-Structured Model for Trachoma Transmission Dynamics, Pathogenesis and Control. PLoS Neglected Tropical Diseases. 2009 6;3(6):1–8.

[5] Grassly NC, Ward ME, Ferris S, Mabey DC, Bailey RL. The Natural History of Trachoma Infection and Disease in a Gambian Cohort with Frequent Follow-Up. PLoS Neglected Tropical Diseases. 2008 12;2(12):1–9.

[6] West ES, Munoz B, Mkocha H, Holland MJ, Aguirre A, Solomon AW, et al. Mass Treatment and the Effect on the Load of Chlamydia trachomatis Infection in a Trachoma-Hyperendemic Community. Investigative Ophthalmology & Visual Science. 2005;46(1):83 – 87.

[7] Solomon AW, Holland MJ, Burton MJ, West SK, Alexander ND, Aguirre A, et al. Strategies for control of trachoma: observational study with quantitative {PCR}. The Lancet. 2003;362(9379):198 – 204.

[8] Solomon AW, Holland MJ, Alexander NDE, Massae PA, Aguirre A, Natividad-Sancho A, et al. Mass Treatment with Single-Dose Azithromycin for Trachoma. New England Journal of Medicine. 2004;351(19):1962–1971.

[9] Liu F, Porco TC, Mkocha HA, Muoz B, Ray KJ, Bailey RL, et al. The efficacy of oral azithromycin in clearing ocular chlamydia: Mathematical modeling from a community-randomized trachoma trial. Epidemics. 2014;6:10 – 17.

[10] Keenan JD, Ayele B, Gebre T, Zerihun M, Zhou Z, House JI, et al. Childhood Mortality in a Cohort Treated With Mass Azithromycin for Trachoma. Clinical Infectious Diseases. 2011;52(7):883–888.

[11] Garthwaite PH, Fan Y, Sisson SA. Adaptive Optimal Scaling of Metropolis-Hastings Algorithms Using the Robbins-Monro Process. ArXiv e-prints. 2010;.
